# Evaluation of the vestibulo-ocular reflex in patients with chronic otitis media

**DOI:** 10.1016/j.bjorl.2020.09.006

**Published:** 2020-10-16

**Authors:** Andreza Tomaz, Rafael da Costa Monsanto, Flavia Salvaterra Cusin, Ana Luiza Papi Kasemodel, Norma de Oliveira Penido

**Affiliations:** a Universidade Federal de São Paulo, Escola Paulista de Medicina (Unifesp/EPM), Departamento de Otorrinolaringologia e Cirurgia de Cabeça e Pescoço, São Paulo, SP, Brazil; b Hospital Israelita Albert Einstein, São Paulo, SP, Brazil

**Keywords:** Vestibulo-ocular reflex, Otitis media, Vestibular function tests, Head impulse test, Semicircular canals

## Abstract

**Introduction:**

In recent years, scientific evidence has shown that chronic otitis media may cause balance and vestibular dysfunction.

**Objective:**

To compare the results of the video head impulse test (gain and symmetry of the vestibulo-ocular reflex and presence of covert and overt saccades) in patients with chronic otitis media and controls.

**Methods:**

Cross-sectional study of patients with chronic otitis media (study group), aged between 18 and 60 years. The patients in the study group were further divided according to the chronic otitis media type as (1) non-suppurative, (2) suppurative, and (3) cholesteatomatous. For the comparative analysis, we selected volunteers with no history of ear and vestibular diseases (control group), who met the same inclusion and exclusion criteria as the study group. Patients in both groups underwent a video head impulse test.

**Results:**

The study group consisted of 96 volunteers, and the control group of 61 individuals. The prevalence of vestibular symptoms was 66% in the study group and 3.2% in the control group (*p* <  0.001). The results show a higher prevalence of changes in the vestibulo-ocular reflex gain (22.9%) and corrective saccades (12.6%) in the chronic otitis media group compared to the control group (*p* <  0.001). Despite the higher prevalence of changes in gain, the average vestibulo-ocular reflex gains in the chronic otitis media groups were within the pre-defined values ​​of normality; however, the mean vestibulo-ocular reflex gain in the anterior semicircular canal was statistically worse in the cholesteatomatous chronic otitis media group compared to controls (*p* <  0.001). Regarding the corrective saccades, the prevalence of saccades was statistically higher in the suppurative and cholesteatomatous chronic otitis media subgroups compared to the non-suppurative and control groups (*p* =  0.004).

**Conclusion:**

The present study demonstrated that chronic otitis media is associated with a higher prevalence of vestibular symptoms and also a higher prevalence of changes in gain and corrective saccades when compared to controls.

## Introduction

Otitis media (OM) is a disease with high rates of incidence and prevalence.^1^ OM and its respective complications and sequelae have significant social, labor and economic impact.^2^, ^3^, ^4^, ^5^, ^6^ Although OM in all its clinical presentations (acute, serous and chronic) develops with varying degrees of lesions in the inner ear, the chronic form (COM) is the one that has been shown to result in a higher prevalence of disabling hearing loss, tinnitus and balance and vestibular alterations.^7^, ^8^, ^9^

In recent years, authors have raised the hypothesis that the vestibular system could also be subject to inflammatory lesions secondary to COM.^3^, ^10^, ^11^, ^12^ These hypotheses were supported by clinical studies, which disclosed a high prevalence of vestibular symptoms in patients with COM (40% –60%), in addition to changes in several vestibular function tests (caloric testing, rotary chair, vestibular evoked myogenic potentials and posturography).^3^

Over the years, several studies have analyzed the potential clinical impact of COM on vestibular function.^3^ However, a recent systematic review^3^ showed that these studies are mostly of low quality and have had several biases identified (selection, attrition, performance and detection). Also, there are a number of difficulties regarding changes in vestibular function secondary to COM,^13^, ^14^ mainly because several vestibular function tests (caloric testing, vestibular evoked myogenic potentials) do not have validated results for patients with middle ear disorders ​​inherent to COM (tympanic perforation, ossicular erosion, fibrosis or secretion in the middle ear, and conductive hearing loss), and therefore are not directly comparable to those of patients without middle ear diseases.^3^, ^13^, ^15^ Furthermore, COM is more prevalent in the elderly, who have several other possible causal factors for vestibular symptoms.^14^, ^16^, ^17^ Therefore, directly correlating COM with changes in vestibular function is a challenging task.^3^

One of the vestibular function tests of that is not significantly influenced by COM is the video head impulse test. This test can objectively assess the function of each of the semicircular canals alone, providing important information regarding changes in the vestibulo-ocular reflex (VOR).^18^, ^19^ Despite these advantages, the literature on the use of the video head impulse test in patients with COM is very scarce. Considering the role of the head impulse test in the diagnosis of VOR alterations, the identification of alterations in this test can help in the topographic characterization of vestibular alterations secondary to COM, and also result in the development of strategies aimed at the prevention of sequelae and selection of adequate treatment and rehabilitation.^3^

## Objective

To compare the results of the video head impulse test (VOR gain and corrective saccades) in patients with COM and controls.

## Methods

### Ethical considerations

When selecting patients and performing the examinations, the ethical, moral and biosafety principles that regulate scientific research with human beings established by the “Declaration of Helsinki” were taken into account. All participants received information about the research content through an explanatory letter and their data were used only after informed consent was provided. Data confidentiality was ensured by assigning an identification number to each patient, which was used to evaluate the results. The data were analyzed in blinded fashion regarding gender, age, and group in which the volunteer was included. As for the performed diagnostic tests, these are not invasive, and do not usually lead to any type of discomfort or risk. This study was approved by the Research Ethics committee of Universidade Federal de São Paulo (UNIFESP), under number 0466/2017.

### Selection of volunteers

A cross-sectional study was carried out with patients diagnosed with COM from the Otology Outpatient Clinic of the Department of Otorhinolaryngology and Head and Neck Surgery (UNIFESP-EPM).

The diagnosis of COM was defined according to the criteria described by Bluestone et al.^20^: presence of chronic middle ear inflammation and/or infection (> 3 months) with tympanic membrane perforation, associated with the presence of clinically intractable tissue alterations in the middle ear (cholesteatoma, granulation tissue, cholesterol granuloma, fibrosis, bone or ossicular erosion, tympanosclerosis). Patients with COM were subdivided into three subgroups^20^: (A) chronic non-suppurative OM, classified as the presence of chronic tympanic membrane perforation caused by otitis media, with otorrhea and infrequent infections (CNSOM); (B) non-cholesteatomatous chronic suppurative OM, defined as the presence of chronic, frequent or intractable otorrhea, which is externalized through tympanic membrane perforation (CSOM); and (C) cholesteatomatous chronic OM, defined as the presence of white or pearly material in the middle ear or mastoid, associated with bone or ossicular chain erosion, with computed tomography also showing suggestive findings (erosion of the spur of Chausse, enlargement of the Prussak space and the *Aditus* ad *Antrum*).

Patients with COM were consecutively selected, using a non-probabilistic convenience sampling method. Patients aged between 18 and 60 years who agreed to participate were included in the study. The exclusion criteria aimed to eliminate patients with possible causes of vestibular symptoms other than COM. Therefore, the exclusion criteria comprised: (A) patients with a history of previous otologic surgery (except insertion of ventilation tubes); (B) patients with cognitive impairments; (C) patients with clinical otosclerosis, definite or probable Ménière’s disease, or definite or probable migraine; (D) previous use of aminoglycosides (oral, parenteral or topical); (E) significant exposure to noise; (F) hematological or head and neck cancers; (G) patients undergoing systemic chemotherapy or radiotherapy of the head and neck region; (H) patients with uncompensated metabolic or cardiovascular disease; (I) diabetes or insulin resistance; (J) autoimmune diseases; (K) neurological or neurodegenerative diseases; and (L) middle ear malformations. Moreover, we excluded patients with severe eye diseases or diseases that made it impossible to properly record the VOR through the video head impulse test. For the diagnosis, identification and consequent exclusion of patients with metabolic and cardiovascular diseases not diagnosed or not adequately treated, all patients underwent an overall physical examination and laboratory tests were requested (fasting glucose, 3-h glucose-insulin curve, lipid profile, thyroid-stimulating hormone [TSH] and free thyroxine [free T4]).

We also selected, based on a non-probabilistic convenience sample, volunteers without ear and vestibular diseases, who were included in a control group. To prevent selection and sampling bias, the selected volunteers mainly consisted of the companions of the people treated at the outpatient clinic where the study group patients were selected. The volunteers were paired as closely as possible in terms of gender and age to the patients included in the COM subgroups (CNSOM, CSOM and cholesteatomatous COM), according to the availability of volunteers who met the inclusion and exclusion criteria.

### Video head impulse test

Patients included in all groups underwent a video head impulse test using the ICS Impulse System equipment (Otometrics A/S Taastrup, Denmark).^21^ This test allows the objective measurement of the semicircular canals (SCC) function through the evaluation of the VOR gain of each SCC alone. The video head impulse test equipment contains goggles with a high resolution video camera (250 Hz) and a transparent mirror, which reflects the image of the patient's right eye to the camera. A small sensor measures the speed of head movements: this movement velocity is captured by the camera and processed by a computer program. The system was firmly attached to the patient's head, to prevent movement of the goggles and reduce the risk of artifacts.

The test was performed according to the classic methodology proposed by MacDougall et al.^18^ After the initial calibration procedure, the number of movements in the direction of each SCC was defined in 20 pulses. When artifacts were identified, the test was repeated once again. All tests were performed by a single researcher with experience in performing the video head impulse test. All individual impulses were assessed for the presence of artifacts by another researcher, also experienced in interpreting this test, who was blinded to demographic information (gender, age) and to which group (experimental or control) the individual belonged.

The software of the ICS Impulse System equipment automatically calculated the mean individual gains of the head impulse by video, in which it is defined by the ratio of the area under the curve for the movement of the eyes under the head movement.^21^

The incidence and amplitudes of the corrective saccades were analyzed. Corrective saccades were considered pathological when they appeared in more than 50% of head impulses. The minimum amplitude of saccades should be at least half the velocity of the head movement, as minor corrective saccades are seen in healthy individuals. The saccades should appear in the opposite direction to that of the head rotation and should occur approximately 100 milliseconds from the beginning to approximately 100 milliseconds after the end of the head movement.^22^, ^23^, ^24^, ^25^, ^26^

The test was considered abnormal when the results of the VOR gain were less than 0.8 (horizontal canals) or 0.7 (vertical canals), in association with the presence of pathological corrective saccades.^18^, ^19^, ^21^, ^23^

### Statistical analysis

For the purpose of comparative analysis of the average gain in each semicircular canal, the ear with COM was considered as the “affected ear” in unilateral cases, and the worse ear from an infectious point of view (higher frequency of otorrhea according to the patient’s perception) in patients with bilateral COM. The results obtained in the “affected ear” of patients with COM were compared with the results obtained in the control group.

The statistical software “SPSS” for Windows (IBM; Armonk, New York, USA) was used for the statistical analysis. Qualitative data were analyzed using frequencies and the chi-square test. For quantitative data, mean, median, standard deviation, variance, skewness and kurtosis were evaluated. According to the distribution (Kolmogorov-Smirnov test), the data were analyzed using parametric (Student's *t* test, ANOVA with Tukey's post-hoc test) or non-parametric tests (Mann-Whitney U test, Kruskal Wallis with Bonferroni-Dunn post-hoc test). Confounding variables such as age were controlled using a linear regression model. The results were considered statistically significant when the *p*-value was < 0.05.

## Results

### Demographic data

The selection of patients with COM resulted in a total of 144 volunteers. Of these, 48 (33.3%) were excluded according to the pre-established criteria. The final group of patients with COM included a total of 96 volunteers. The control group consisted of 61 individuals. Demographic data are described in Table 1.

**Table 1:** Demographic information of volunteers in the control and COM groups.

Demographic data	Controls	COM (total)	CNSOM	CSOM	Cholesteatomatous COM
Number of volunteers	61 (100%)	96 (100%)	43 (45%)	22 (23%)	31 (32%)
Mean / SD age (years)	36.1 ± 12.7	44.6 ± 16.0	44.7 ± 15.4	47.9 ± 16.2	40.0 ± 15.8
Gender	F/ 46 (75%)	F/ 67 (70%)	F, 28 (65%)	F/ 17 (77%)	F/ 22 (71%)
Unilateral disease	–	61 (64%)	28 (65%)	13 (59%)	20 (65%)
Bilateral disease	–	35 (36%)	15 (35%)	9 (41%)	11 (35%)
Dizziness-mean	3.2%	66%	53%	86%	68%

COM, Chronic Otitis Media; CNSOM, Chronic Non-Suppurative Otitis Media; CSOM; Chronic Suppurative Otitis Media; SD, Standard Deviation.

The statistical analysis of demographic data showed no significant differences in relation to age between the groups (*p* > 0.05), except when comparing the CSOM (± 47.9 years) and control groups (36.1 ± 12.7) (*p* =  0.022) (Table 1). We did not observe significant differences between the gender distribution between the control groups and COM subgroups (*p* =  0.64).

### Anamnesis

At the initial evaluation, 66% of patients in the total COM group complained of vestibular symptoms. When comparing the COM subgroups, there was a higher prevalence of dizziness in the CSOM Group (86%) and cholesteatomatous COM (68%) than in the Control Group (3.2%) and CNSOM (53%) (*p* <  0.001).

We did not observe the presence of spontaneous nystagmus with visual fixation in any of the volunteers in the COM and control groups. In the clinical head impulse test, corrective saccades were present in 3% of patients in the COM group.

### Video head impulse test

The mean value, standard deviation, variance, skewness and kurtosis of the VOR gain in the SCC of the COM and control groups are shown in Table 2.

**Table 2:** Mean gain, standard deviation, variance, skewness and kurtosis of the vestibulo-ocular reflex obtained in the semicircular canals tested in the different groups with the video head impulse test.

		Anterior SCC	Lateral SCC	Posterior SCC
Controls	Mean	0.91	0.95	0.82
	Standard deviation	0.06	0.07	0.04
	Variance	0.004	0.005	0.002
	Skewness	−0.261	0.107	0.184
	Kurtosis	−0.485	0.608	0.256
CNSOM	Mean	0.86	0.94	0.78
	Standard deviation	0.12	0.08	0.08
	Variance	0.015	0.007	0.007
	Skewness	−0.526^a^	0.270	0.410
	Kurtosis	0.742	−0.324	0.713
CSOM	Mean	0.9	0.97	0.82
	Standard deviation	0.86	0.11	0.12
	Variance	0.015	0.013	0.015
	Skewness	−1.184^b^	0.150	−1.452^b^
	Kurtosis	1.670	1.254	3.523
Cholesteatoma	Mean	0.82	0.99	0.78
	Standard deviation	0.11	0.11	0.12
	Variance	0.013	0.013	0.015
	Skewness	−0.037	−0.248	−0.662^a^
	Kurtosis	0.409	−0.056	1.482

CNSOM, Chronic Non-Suppurative Otitis Media; CSOM, Chronic Suppurative Otitis Media; Cholesteatoma, Cholesteatomatous Chronic Otitis Media; SCC, Semicircular Canal.

a Moderately skewed distribution value (0.5–1.0).

b Highly skewed distribution value (> 1.0).

After processing the results of the head impulse test, it was observed that there were no abnormalities in the test (reduced gain associated with the presence of corrective saccades) in any of the volunteers from the control group. In the COM group, the test was considered normal in 84 (87.5%) of the patients, while in the other 12 (12.5%) the test showed a reduction of the VOR gain in at least one semicircular canal with the presence of corrective saccades.

**Figure 1:**
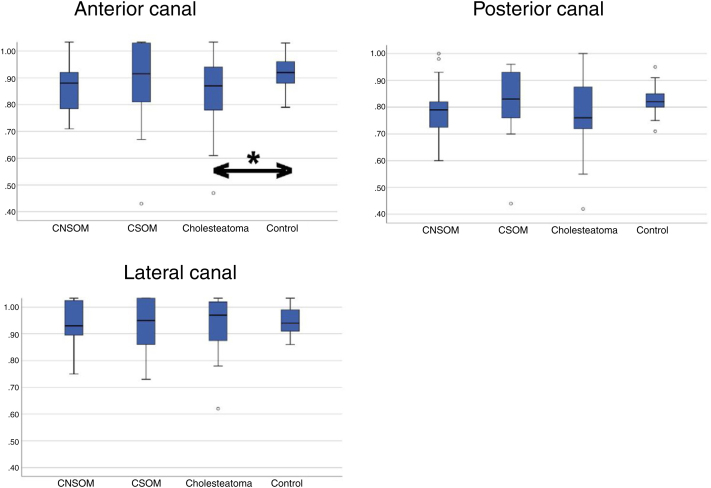
Distribution of the results obtained in each semicircular canal on the affected/worst side of the chronic otitis media subgroups compared to the results obtained in the control group. CNSOM, Chronic Non-Suppurative Otitis Media; CSOM, Chronic Suppurative Otitis Media; Cholesteatoma, Cholesteatomatous Chronic Otitis Media. *Statistically significant difference (*p* < 0.05).

The mean results of the VOR gain in the anterior, lateral and posterior SCCs of the control and study groups were within the pre-defined values ​​of normality (0.8 for lateral canals, 0.7 for anterior and posterior canals) (Table 2). Analyzing the results of each patient individually, we observed a decrease in the VOR gain (as compared with normative data) in at least one of the SCCs (22.9%) of the patients from the COM group. The decrease in VOR gain seen in these 22 patients was associated with the presence of corrective saccades in 12 (54.5%) of these patients. In the control group, no decrease in the VOR gain was seen in any of the volunteers in our sample, which was statistically significant compared to that observed in the COM Groups (*p* <  0.001). There was also greater variability in the distribution of results obtained in the COM Groups (skewness variation = 0.037–1,452) compared to the control group (skewness variation = 0.107–0.261). The variability of results between the COM and controls groups was significant for the anterior (F = 6,128; *p* =  0.001) and posterior SCCs (F = 2,992; *p*  = 0,033), but not for the lateral SCCs (F = 2,012; *p* =  0.115). Although, on average, the results of the VOR gain are within the pre-defined normal values, it was observed that the anterior SCC gain in the cholesteatomatous COM subgroup (mean = 0.82; standard deviation – SD = 0.11) was significantly lower compared to that obtained in the control group (mean = 0.91; SD = 0.06) (*p* <  0.001) (Fig. 1, Table 3).

**Table 3:** Comparison (p-value) between the mean gain of the lateral, anterior and posterior SCC of the affected/worse ear COM subgroups and of the control group volunteers in the video head impulse test.

		Anterior SCC (P)	Lateral SCC (P)	Posterior SCC (P)
Controls	CNSOM	0.054	0.842	0.063
	CSOM	0.164	0.867	0.997
	Cholesteatomatous	< 0.001^a^	0.305	0.135
CNSOM	CSOM	1	0.526	0.317
	Cholesteatomatous	0.35	0.096	0.063
CSOM	Cholesteatomatous	0.515	0.9	0.412

COM, Chronic Otitis Media; CNSOM, Chronic Non-Suppurative Otitis Media; CSOM, Chronic Suppurative Otitis Media; SCC, Semicircular Canal.

a *p* <  0.005.

Regarding the corrective saccades, the video head impulse test showed the presence of corrective saccades in 12 (12.5%) of the patients, all of which also showed an associated decrease in the VOR gain in the affected SCC. The prevalence of corrective saccades was higher in patients with COM (12.5%) as compared to the control group (0.0%) (*p* < 0.001). Regarding the COM subgroups and controls, the results showed a higher prevalence of corrective saccades in the CSOM (18.1%) and cholesteatomatous COM (16.1%) groups compared to the CNSOM (4.6%) and control groups (0 %) (*p* =  0.004). Corrective saccades were observed in the lateral SCCs (6 patients with COM) and posterior SCCs (6 patients with COM); we did not observe the presence of corrective saccades during the test of the anterior SCCs.

## Discussion

In the present study, the results showed a higher prevalence of changes in the results of the video head impulse test in patients with different types of COM in comparison to the control group. The present study comprises the largest sample (regarding the number) of patients with COM (CNSOM, CSOM and cholesteatomatous OM), submitted to the video head impulse test, providing the best scientific evidence available in the literature on the subject. The video head impulse test has a number of advantages in assessing the vestibular function of patients with COM,^27^ since it is an easily validated test,^18^, ^19^ not negatively influenced by the patient’s age 28, 29 or due to the presence of conductive or mixed hearing loss.^30^

The analysis of demographic data showed that the mean age of one of the COM subgroups (CSOM) was statistically higher in relation to the control group. This age difference could be one of the factors that led to a higher prevalence of vestibular symptoms in the CSOM group compared to the control group, since vestibular symptoms are more frequent in the population over 40 years old. 14, 16, 18, 28^,^^29^, ^31^ However, the methodology applied in this study and some of the findings minimize the impact of potential age differences on the assessment. The exclusion criteria used in the study, although quite restrictive, aimed at (1) reducing the risk of selection and sampling bias; and (2) exclude several possible underlying causes that could lead to the presence of these vestibular symptoms; these include decompensated metabolic and cardiovascular diseases, which are the main sources of vestibular problems in this age group.^17^ Additionally, the patients included in our sample (both in the COM groups and in the control group) are mostly in the same age group (fourth and fifth decades of life), which reduces the impact of age on the analyses. In the other two groups of COM (CNSOM and cholesteatomatous COM), there were no statistically significant differences in relation to the mean age in comparison to the control group, which suggests that COM can show a higher prevalence of vestibular symptoms regardless of age. Moreover, studies have shown that age is not a factor that can, by itself, generate significant changes in the results of the VOR gain and the presence of corrective saccades in the video head impulse test.^28^, ^29^ Even considering these observations, we used a linear regression model with age as a covariate in the analysis of the clinical test results of vestibular function and in the head impulse test to reduce the hypothetical impact of age on these evaluations.

In relation to the video head impulse test, we identified a higher prevalence of corrective saccades in all COM subgroups (variation between groups, 4.6% –18.1%) when compared to controls (0.0%) (*p* <  0.001). The comparative analysis between the COM subgroups showed that the prevalence of saccades in the CSOM and cholesteatomatous COM groups was significantly higher compared to that found in the CNSOM group (*p* = 0.004). Regarding the mean VOR gain of the SCCs, the obtained mean in all the tested canals was within the parameters of normality, even in the COM groups; however, there was a statistical difference in the means obtained in the anterior SCC in the cholesteatomatous group compared to the control group (*p* < 0.001), while the rest of the comparisons between groups and different canals did not reveal statistically significant differences (*p* > 0.05). Despite the absence of changes in the absolute values ​​of the VOR mean in the COM groups, some data suggest that COM can produce detectable alterations in the video head impulse test in some patients with COM. Among these alterations, the following stand out: (1) The greater variability and asymmetry of the VOR gain results in patients with COM compared to controls; (2) There was a higher prevalence of alterations in gain (compared to normative data) in the COM group compared to the control group; and (3) There was a higher prevalence of corrective saccades (associated with the presence of reduced VOR gain) in patients with COM compared to the control group. Therefore, it is possible that the results show possible changes in the SCC function secondary to COM, even in cases in which there are no associated corrective saccades. Although the findings support this hypothesis, to date, there is no experimental study in the literature that supports the hypothesis that COM causes direct injuries to the peripheral vestibular system, especially to SCCs. Despite this, histopathological studies in temporal bones with COM revealed a significant reduction in the density of hairy cells type I and II, in the macules of the saccule and utricle, and hairy cells type I in the ampullary crest of the lateral and posterior semicircular canals of temporal bones with COM in compared to controls.^10^, ^11^, ^32^, ^33^ Therefore, it is possible that the findings of these histopathological studies explain at least partially the changes in gain found in the present study.

In the literature, research dedicated to assessing the results of the video head impulse test in patients with COM is scarce.^3^ In a case study of three patients with radiologically and surgically confirmed horizontal SCC fistula secondary to COM, D’Albora et al.^34^ reported that the head impulse test at the bedside (without video) revealed the presence of refixation saccades in the three patients. Additionally, two of these patients underwent a video head impulse test, which showed a decrease in the VOR gain with consequent covert and overt saccades. In 2019, Covelli et al.^35^ evaluated, through the video head impulse test, the lateral SCC function in the pre- and postoperative period of 8 patients with cholesteatomatous COM with labyrinthic fistula. These authors observed, in the preoperative exam, results of normal VOR gain in 62.5% of the cases and suggested that the simple labyrinth exposure due to bone erosion is unable to induce a functional impairment if an additional cause, such as a toxic effect, is not concomitant. Additionally, they report that although a limited number of subjects were evaluated, this study allowed the assessment of vestibular function in the case of lateral SCC fistula and helped to obtain preoperative information about SCC function when symptoms are minimal and non-instrumental signs are negative, although no statistical validation has been obtained.

The present study has limitations. The applied exclusion criteria were very restrictive, decreasing the number of patients in the study. However, this was necessary to reduce possible biases as much as possible, as the objective would be compromised if these criteria were not used. As reported by other studies,^17^ there is a high prevalence of vestibular symptoms even in patients considered “healthy”, which may also have impacted the results of the present study. To minimize the impact of this fact on our results, patients were selected consecutively, and underwent a systematic routine of clinical and laboratory evaluation carried out by otologists and neurotologists in order to exclude, as accurately as possible, the presence of other factors that could act as confounding factors. Another limitation is that the video head impulse test, although not directly affected by the presence of alterations in the middle ear and conductive or mixed hearing loss, has other limitations, mainly with regard to its role in the diagnosis of chronic or compensated vestibular disorders.^27^, ^30^ However, the present study provides very relevant information from a clinical point of view and opens the door for future research in this regard. It is possible that the use of the video head impulse test may, in the future, become an important tool for the evaluation of the progression of vestibular lesions secondary to COM. Another point that can be further studied in the future is the use of this test in the early evaluation of acute complications in patients with COM, such as suppurative labyrinthitis. Moreover, it is possible that future clinical studies may, with a more accurate assessment of the patients’ symptoms and associated with the results of other vestibular function tests, more accurately demonstrate which are the risk factors in patients with COM regarding the development of changes in the video head impulse test identified in this study.

## Conclusion

The results of this study demonstrate a higher prevalence of changes in gain (22.9%) and corrective saccades (12.6%) in the video head impulse test in patients COM as compared with controls.

## Funding

Three authors (AT, RCM, and ALPK) received a scholarship from the “Coordenação de Aperfeiçoamento de Pessoal de Nível Superior, Brazil (CAPES)” (Finance Code 001).

## Conflicts of interest

The authors declare no conflicts of interest.
